# Effects of Nonextensive Electrons on Dust–Ion Acoustic Waves in a Collisional Dusty Plasma with Negative Ions

**DOI:** 10.3390/e25091363

**Published:** 2023-09-21

**Authors:** Zhipeng Liu

**Affiliations:** School of Science, Tianjin Chengjian University, Tianjin 300384, China; zhipengliu@tcu.edu.cn; Tel.: +86-22-23085304

**Keywords:** ion acoustic waves, nonextensive statistics, kappa distribution, Korteweg–de Vries Burgers equation

## Abstract

The effects of nonextensive electrons on nonlinear ion acoustic waves in dusty negative ion plasmas with ion–dust collisions are investigated. Analytical results show that both solitary and shock waves are supported in this system. The wave propagation is governed by a Korteweg–de Vries Burgers-type equation. The coefficients of this equation are modified by the nonextensive parameter *q*. Numerical calculations indicate that the amplitude of solitary wave and oscillatory shock can be obviously modified by the nonextensive electrons, but the monotonic shock is little affected.

## 1. Introduction

Ion acoustic wave (IAW) is a low-frequency electrostatic wave that can be commonly observed in space and experimental plasmas. Its linear or nonlinear properties have long been studied in the past decades. Examples include Landau damping [1,2], IAW instabilities [3,4], solitary wave propagation [5,6], etc. Among the above wave phenomena, ion acoustic solitary and shock wave problems occupy an important place in studies of plasmas. Early research on IAW can be traced back to the 1960s. Biscamp et al. theoretically investigated the shock structure and formation of IAW in a collisionless plasma [7]. They found that the wave can be described by a Korteweg–de Vries Burgers (KdVB) equation. For the first time, Ikezi et al. observed the shock wave structure in a novel double-plasma device [8]. Das gave systematic studies on IA solitary and shock waves in plasmas with negative ions [9]. Since then, explorations of IAW in muti-components plasmas have attracted much attention. The pioneering works by Shukla and Silin showed that IAW could also be supported in dusty plasma, namely dust–ion acoustic wave (DIAW) [10]. The dust grains, which have micrometer or sub-micrometer sizes, are ubiquitous in space and laboratory environments, such as solar wind [11], planetary rings [12], the interstellar medium [13], the Earth’s lower ionosphere [14], semiconductor processing devices [15] and fusion plasmas [16]. A number of authors have shown that the existence of charged dust grains could modify the dynamical behavior of electrostatic waves in plasmas. These modifications may be due to the charge variation [17,18], dust size distribution [19], dust density waves [20], temperature [1,21], etc. Therefore, the wave properties in dusty plasmas would be quite complicated but interesting, especially for the coherent structure of IAW. Meanwhile, numerical simulation have shown that particle distributions of fluid systems, such as multiphase flows [22] and high-speed compressible flow [23,24,25], usually deviate from Maxwellin distribution. As a typical fluid system, plasma usually exhibits a power-law form distribution and cannot be modeled by Maxwellian distribution [26,27,28]. For instance, data from spacecraft or laboratory plasmas observations often reveal that plasmas often process a number of superthermal electrons (energetic particles). These high-energy particles make the plasmas obviously deviate from the Maxwellian. Vasyliunas was the first to give an empirical power-law form expression called generalized Lorentzian (kappa) distribution to model these superthermal particles [29]. They found that the plasmas can be well fitted by kappa distribution. Recently, Leubner [30], Livadiotis and McComas [31,32,33] have theoretically proven that the kappa-type distributions are a consequence of Tsallis distribution in nonextensive statistics [34]. Nonextensive statistics was first introduced by Tsallis [34] and further developed by many others. In nonextensive statistics, the nonadditive *q*-entropy has the form,

(1) $$S_{q}=k_{B}\frac{\int \left(f^{q}-f\right)dx^{3}dv^{3}}{1-q},$$

where *f* is the probabilistic distribution function, and *q* is a real parameter different from unity, specifying the degree of nonextensivity. It was proven that *q* is related to the temperature gradient and the gravitational potential [35]. The physical meaning of *q* is connected to the non-isothermal (nonequilibrium stationary state) nature of the systems with long-range interactions.

Nowadays, nonextensive statistics have successfully been applied to a number of systems [36,37] and become a powerful tool to analyze complex systems with Coulomb long-range [38], self-gravitating interactions [39,40], astrophysics [41] and plasma physics phenomena such as ion acoustic instability [26], dust acoustic instability [27], permeating plasmas [42,43], transport [44], diffusion [45], viscosity [46], and statistical uncertainty [47] effects. For plasma waves, examples could be numerous. For instance, Lima et al. discussed the dispersion relations and Landau damping for electrostatic plane–wave propagation in a collisionless thermal plasma in the context of nonextensive statistics [48]. Tribeche et al. explored arbitrary amplitude ion acoustic solitary waves in a two-component plasma with a nonextensive electron velocity distribution. Their results showed that the ion acoustic solitary wave amplitude was sensitive to the nonextensive parameter *q* [49]. EI-Awady and Moslem studied the generation of nonlinear ionacoustic rogue and solitary waves in a plasma with nonextensive electrons and positrons [50]. The results from their work show a dependence of both solitary and rogue wave profiles on the nonextensive parameter. Recently, Yasmin et al. analyzed the modification of DIA shock waves in an unmagnetized, collisionless, dissipative dusty plasma containing nonextensive electrons [51]. They found that shock compression and rarefaction are sensitive to the degree of the nonextensivity of electrons.

Former studies on wave properties in nonextensive plasmas usually assume the plasmas are collisionless. This is reasonable for dustless plasmas, as collisions between ions and electrons are rare. However, when dust grains are encountered in plasmas, due to the large size of dust grains, the collisional effects of ions/electrons with dust grains may not be neglected. Recently, Misra et al. proved that, in a Maxwellian dusty plasma with negative ions, ion–dust collisions play a crucial role in the dissipation of ion acoustic solitary wave and shocks (IASWS) propagation [52]. Therefore, the nonlinear wave structure of non-Maxwellian plasmas, which have not been investigated before, would be very interesting and worth exploring. The aim of the present paper is to investigate the nonextensivity of electrons on IASWS in multi-ion plasma with ion–dust collisions. The paper is arranged as follows: In Section 2, basic equations for describing the system are given. In Section 3, following the standard reductive perturbation method, a KdV Burgers type equation are obtained. In Section 4, numerical calculations with related parameters and the nonextensive index *q* are carried out to check the nonextensivity of electrons on IASWS. Finally in Section 5, the summary and conclusive remarks are given.

## 2. Governing Equations

In this paper, we consider a fully ionized one-dimensional, unmagnetized collisional dusty plasma consisting of nonextensive electrons, positive and negative cold fluid ions, and immobile dust grains. The charge neutrality condition gives:

(2) $$n_{p0}-n_{i0}-n_{e0}\pm Z_{d}n_{d0}=0,$$

where $n_{j0}$ is the unperturbed number density of species *j* (*j* stands for the electrons, dust grains, and positive and negative ions respectively), $Z_{d}$ is the charge number of dust particles, the sign ± before $Z_{d}$ represents the positively (negatively) charged dust. If we let $\mu_{e}=n_{e0}/n_{n0}$, $\mu_{d}=Z_{d0}n_{d0}/n_{n0}$ and $\mu_{i}=n_{p0}/n_{n0}$, then Equation (2) can be written as,

(3) $$\mu_{i}-\mu_{e}\pm \mu_{d}-1=0.$$

The basic equations for describing the dynamics of one-dimensional plasma systems are the following:

(4) $$\begin{array}{c} \frac{\partial n_{j}}{\partial t}+\frac{\partial }{\partial x}\left(n_{j}V_{j}\right)=0, \end{array}$$

(5) $$\begin{array}{c} \left(\frac{d}{dt}+\nu_{jd}\right)V_{j}=-\frac{Q_{j}}{m_{j}}\frac{\partial \phi }{\partial x}-\frac{3k_{B}T_{j}}{m_{j}n_{j0}^{2}}\cdot \frac{\partial n_{j}^{2}}{\partial x}+\eta_{j}\frac{\partial^{2}V_{j}}{\partial x^{2}}, \end{array}$$

(6) $$\begin{array}{c} \frac{\partial^{2}\phi }{\partial x^{2}}=4\pi e\left(n_{e}-n_{p}+n_{n}\mp Z_{d}n_{d}\right), \end{array}$$

where $n_{j}$, $V_{j}$, $Q_{j}$, $m_{j}$ and $T_{j}$ are the number density, velocity, mass, charge and temperature of *j*-species ions, respectively; ϕ is the electrostatic potential; $\nu_{jd}$ is the collision rate of *j*-species ions with dust particles; $\eta_{i}$ is the viscosity coefficient due to ion–dust collisions; and $k_{B}$ is the Boltzmann constant. For simplicity, let us introduce the following dimensionless physical quantities:

$$\begin{array}{c} {\bar{\eta }}_{j}\to \eta_{j}/\lambda_{D}^{2}\omega_{pd},{\bar{\nu }}_{jd}\to \nu_{jd}/\omega_{pd},\phi \to e\phi /k_{B}T_{e},\\ n_{j}\to n_{j}/n_{j0},V_{j}\to V_{j}/c_{s},x\to x/\lambda_{D},t\to t\cdot \omega_{pd} \end{array}$$

where $\lambda_{D}=\sqrt{k_{B}/4\pi Z_{d}n_{d0}e^{2}}$ is the Debye length, $\omega_{pj}=\sqrt{4\pi n_{n0}e^{2}/m_{j}}$ is the plasma frequency, $c_{s}=\sqrt{Z_{d}k_{B}T_{e}/m_{d}}$ is the thermal speed, $\sigma_{j}=T_{j}/T_{e}$ and $\beta_{j}=m_{n}/m_{j}$. The nondimensional form of Equations (4)–(6) become,

(7) $$\begin{array}{c} \frac{\partial n_{j}}{\partial t}+\frac{\partial }{\partial x}\left(n_{j}V_{j}\right)=0, \end{array}$$

(8) $$\begin{array}{c} \left(\frac{d}{dt}+{\bar{\nu }}_{jd}\right)V_{j}=-\beta_{j}\left(\frac{\partial \phi }{\partial x}+\frac{3}{2}\sigma_{j}\frac{\partial n_{j}^{2}}{\partial x}\right)+{\bar{\eta }}_{j}\frac{\partial^{2}V_{j}}{\partial x^{2}}, \end{array}$$

(9) $$\begin{array}{c} \frac{\partial^{2}\phi }{\partial x^{2}}=Z_{d}n_{d}+\mu_{e}n_{e}-\mu_{i}n_{p}+n_{n}. \end{array}$$

We assume that the electrons in the plasma obey the normalized nonextensive electron distribution:

(10) $$n_{e}={\left[1+\left(q-1\right)\phi \right]}^{\left(q+1)/2(q-1\right)},$$

where *q* is the nonextensive parameter that describes the nonextensivity of the electrons.

## 3. Derivation of the KdV Burgers Type Equation

Following the routing procedure, we employ the standard reductive perturbation technique to derive the evolution equation for DIAW. First, let us introduce the new variables of space and time:

(11) $$\xi =\varepsilon^{1/2}\left(x-U_{0}t\right),\tau =\varepsilon^{3/2}t,$$

where ε is a small parameter characterizing the strength of the nonlinearity, $U_{0}$ is the wave speed in the moving frame of reference. We also introduce ${\bar{\nu }}_{jd}=\varepsilon^{3/2}{\bar{\nu }}_{j0}$ and $\eta_{jd}=\varepsilon^{1/2}\eta_{j0}$, where ${\bar{\nu }}_{j0}$ and $\eta_{j0}$ are of the order of unity or less. Next, we expand the dynamical variables as

(12) $$\begin{array}{c} n_{j}=1+\varepsilon n_{j}^{\left(1\right)}+\varepsilon^{2}n_{j}^{\left(2\right)}+\cdots , \end{array}$$

(13) $$\begin{array}{c} V_{j}=\varepsilon V_{j}^{\left(1\right)}+\varepsilon^{2}V_{j}^{\left(1\right)}+\cdots , \end{array}$$

(14) $$\begin{array}{c} \phi =\varepsilon \phi^{\left(1\right)}+\varepsilon^{2}\phi^{\left(2\right)}+\cdots . \end{array}$$

Then we substitute Equations (12)–(14) into Equations (7)–(9) and equate the terms of the same powers of ε. From the $\varepsilon^{3/2}$ terms, we have,

(15) $$n_{j}^{\left(1\right)}=\alpha_{j}\phi^{\left(1\right)},V_{j}^{\left(1\right)}=\alpha_{j}U_{0}\phi^{\left(1\right)},$$

where $\alpha_{j}=\pm \beta_{j}/\left(U_{0}^{2}-3\beta_{j}\sigma_{j}\right)$. Here, the sign ± corresponds to the positive and negative ions, respectively. $U_{0}$ has the form of

(16) $$\begin{array}{c} U_{0}^{2}=\frac{1}{2\left(q+1\right)\mu_{e}}\left\{s\pm \sqrt{s^{2}-12\left(1+q\right)\beta \mu_{e}\left[2\sigma_{p}+3\left(1+q\right)\mu_{e}\sigma_{p}\sigma_{n}+2\mu_{i}\sigma_{n}\right]}\right\}, \end{array}$$

where

(17) $$s=2+2\beta \mu_{i}+3\left(1+q\right)\left(\sigma_{n}+\beta \sigma_{p}\right)\mu_{e}.$$

The ± sign in Equation (16) indicates that there are two values. This means that the plasmas contain two types of ion acoustic waves, the fast mode (+) and the slow one (−). Detailed discussions related to these two modes are given in Section 4. Now, we proceed to the next order of ε, and the following equations for the second order perturbed quantities are obtained:

(18) $$\begin{array}{c} \alpha_{j}\frac{\partial \phi^{\left(1\right)}}{\partial \tau }-U_{0}\frac{\partial n_{j}^{\left(2\right)}}{\partial \xi }+\alpha_{j}^{2}U_{0}\frac{\partial {\left[\phi^{\left(1\right)}\right]}^{2}}{\partial \xi }+\frac{\partial V_{j}^{\left(2\right)}}{\partial \xi }=0, \end{array}$$

(19) $$\begin{array}{c} \left(\frac{\partial }{\partial \tau }+{\bar{\nu }}_{j0}\right)\alpha_{j}U_{0}\phi^{\left(1\right)}+\frac{1}{2}\alpha_{j}^{2}\left(U_{0}^{2}+3\beta_{j}\sigma_{j}\right)\frac{\partial {\left[\phi^{\left(1\right)}\right]}^{2}}{\partial \xi }\\ =U_{0}\left[\frac{\partial V_{j}^{\left(2\right)}}{\partial \xi }+{\bar{\eta }}_{j0}\alpha_{j}\right]\frac{\partial^{2}\phi^{\left(1\right)}}{\partial \xi^{2}}-\beta_{j}\left[3\sigma_{j}\frac{\partial n_{j}^{\left(2\right)}}{\partial \xi }\pm \frac{\partial \phi^{\left(2\right)}}{\partial \xi }\right], \end{array}$$

(20) $$\begin{array}{c} \frac{\partial^{3}\phi^{\left(1\right)}}{\partial \xi^{3}}=\frac{\partial n_{n}^{\left(2\right)}}{\partial \xi }-\mu_{i}\frac{\partial n_{p}^{\left(2\right)}}{\partial \xi }+\mu_{e}\left(\frac{1+q}{2}\right)\left[\frac{\partial \phi^{\left(2\right)}}{\partial \xi }+\frac{3-q}{4}\cdot \frac{\partial {\left(\phi^{\left(1\right)}\right)}^{2}}{\partial \xi }\right]. \end{array}$$

Putting Equation (14) into Equations (18)–(20) and eliminating the second-order quantities, we obtain the following KdVB-type equation:

(21) $$\frac{\partial \Phi }{\partial \tau }+A\Phi \frac{\partial \Phi }{\partial \xi }+B\frac{\partial^{3}\Phi }{\partial \xi^{3}}-\eta \frac{\partial^{2}\Phi }{\partial \xi^{2}}+\nu \Phi =0,$$

where we set $\Phi =\phi^{\left(1\right)}$. The coefficients *A*, *B*, η and ν, which represent the nonlinearity, dispersion, dissipation due to ion kinematic viscosities and ion–dust collisions, respectively, can be written as,

(22) $$\begin{array}{c} A=\frac{3\alpha_{p}^{3}\mu_{i}\left(U_{0}^{2}+\beta \sigma_{p}\right)+3\alpha_{n}^{3}\beta \left(U_{0}^{2}+\sigma_{n}\right)+\beta \left(\frac{3-q}{2}\right)\left(\alpha_{n}-\mu_{i}\alpha_{p}\right)}{2U_{0}\left(\beta \alpha_{n}^{2}+\alpha_{p}^{2}\mu_{i}\right)}, \end{array}$$

(23) $$\begin{array}{c} B=\frac{\beta }{2U_{0}\left(\beta \alpha_{n}^{2}+\alpha_{p}^{2}\mu_{i}\right)}, \end{array}$$

(24) $$\begin{array}{c} \eta =\frac{{\bar{\eta }}_{n0}\alpha_{n}^{2}\beta +{\bar{\eta }}_{p0}\alpha_{p}^{2}\mu_{i}}{2\left(\beta \alpha_{n}^{2}+\alpha_{p}^{2}\mu_{i}\right)}, \end{array}$$

(25) $$\begin{array}{c} \nu =\frac{{\bar{\nu }}_{n0}\alpha_{n}^{2}\beta +{\bar{\nu }}_{p0}\alpha_{p}^{2}\mu_{i}}{2\left(\beta \alpha_{n}^{2}+\alpha_{p}^{2}\mu_{i}\right)}. \end{array}$$

One may see that the evolution of Equation (21) has the same form as obtained by Misra et al. [52]. However, the coefficients *A*, *B*, η and ν, which determine the formation and evolution of ion acoustic wave structures, are modified by the nonextensive parameter *q*. It can be verified that in the limit q→1, the Maxwellian counterparts of these coefficients will be recovered [52]. The effects of these modifications due to nonextensivity will be analyzed in Section 4.

## 4. Numerical Results and Discussion

Equation (21) is a modified KdV Burgers equation that describes the DIAW in a collisional dusty plasma. The effects of the coefficients *A*, *B*, η and ν on the wave evolution for Maxweillian plasmas were discussed by Misra et al. in detail [52]. Therefore, here we just investigate the effects of the nonextensivity of the system. Since the exact solution of Equation (21) is still unknown, in order to obtain the effects of the nonextensivity of the wave evolution, we numerically investigate the influences of the nonextensive parameter *q* on the coefficients *A*, *B*, η and ν, respectively. During our calculation, the following space and laboratory observed parameters are employed (see Reference [52] for more details) for negatively charged dust, $m_{n}=146m_{proton}$, $m_{n}=39m_{proton}$, $T_{e}∼T_{p}∼0.2$ eV, $T_{n}∼T_{e}/8$, $n_{n0}∼2\times 10^{9}$, $\eta_{p0}=0.3$, $\eta_{n0}=0.5$, $\nu_{p0}=0.01$, $\nu_{p0}=0.01$, where $m_{proton}$ is the mass of protons. For positively charged dust, $m_{n}=146m_{proton}$, $m_{n}=39m_{proton}$, $T_{e}∼T_{p}∼0.2$ eV, $T_{n}∼T_{e}/2$, $n_{n0}∼2\times 10^{9}$, $\eta_{p0}=0.5$, $\eta_{n0}=0.3$, $\nu_{p0}=0.5$, and $\nu_{p0}=0.3$.

**Figure 1:**
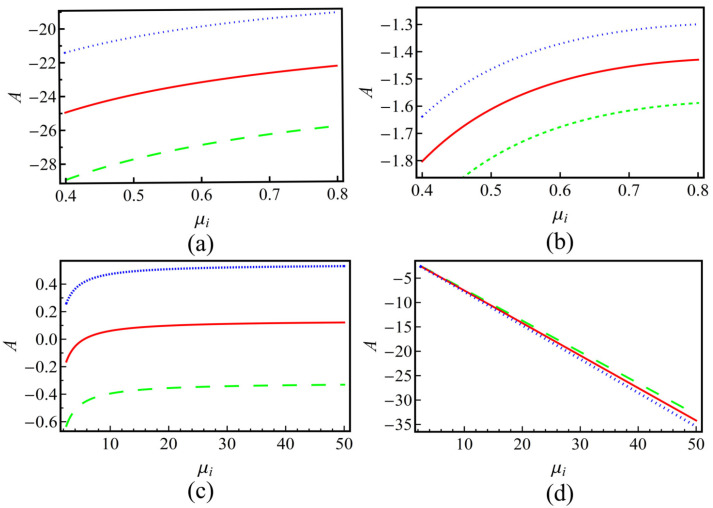
The nonlinearity coefficient *A* versus the positive-to-negative ion density ratio $\mu_{i}$ for plasmas with positive (subplots (**a**,**b**)) and negative (subplots (**c**,**d**)) charged dusts. The left panels (subplots (**a**,**c**)) corresponding to the positive sign in ${U_{0}}^{2}$ (in Equation (16)) and the right ones (subplots (**b**,**d**)) are negative. Lines in each subplot represent different nonextensive *q* values, where the blue dotted lines represent q=1.2, the red solid lines represent q=1.0 and the green dashed lines represent q=0.8, respectively.

Figure 1 shows that the nonlinearity coefficient *A* varies with $\mu_{i}$ for positively and negatively charged dust, respectively. As shown in Figure 1, with increasing $\mu_{i}$, the strength of *A* will increase in subplots (a)–(c) but decrease in subplot (d). We can also obtain that if *A* has a growing trend, with fixed $\mu_{i}$, the sub-extensive case (q>1) has the largest value, while the super-extensive case (q<1) has the smallest one. If *A* has a decreasing trend [subplot (d)], the nonextensive effects are opposite to those of (a)–(c), then the subextensive case (q<1) has the largest value. Therefore, the nonextensivity of the system has an enhancement on the growth or decrease in *A*.

**Figure 2:**
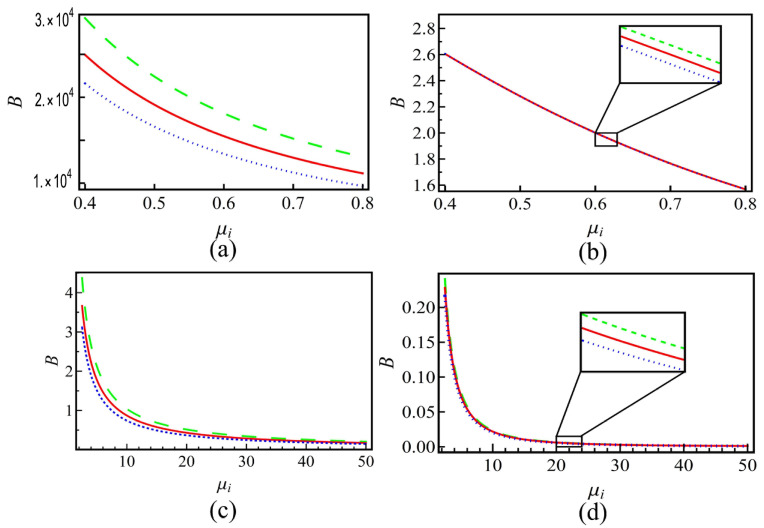
The nonlinearity coefficient *B* versus the positive-to-negative ion density ratio $\mu_{i}$ for plasmas with positive (subplots (**a**,**b**)) and negative (subplots (**c**,**d**)) charged dusts. The left panels (subplots (**a**,**c**)) corresponding to the positive sign in ${U_{0}}^{2}$ (in Equation (16)) and the right ones (subplots (**b**,**d**)) are negative. Lines in each subplot represent different nonextensive *q* values that are the same as that in Figure 1.

Figure 2 gives that the dispersion coefficient *B* varies with the ion density ratio $\mu_{i}$. From the figure, it is found that *B* will monotonically decrease with the growth in $\mu_{i}$. The left panels (subplots (a) and (c)) indicate that the nonextensive effects on *B* are obvious. In the right panels (subplots (b) and (d)), it is found that the three lines are nearly overlapped. In this case, the effect of nonextensivity is quite weak. Amplification of the curves shows that with the growth of $\mu_{i}$, the system nonextensivity has enhancement on the growth/decrease in the dispersion coefficient *B*. Therefore, the effects of nonextensivity are the same as those of *A*.

**Figure 3:**
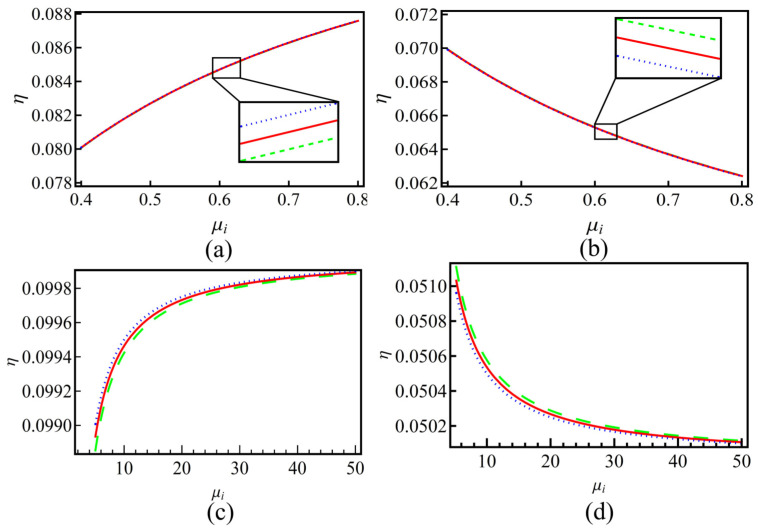
The nonlinearity coefficient *B* versus the positive-to-negative ion density ratio $\mu_{i}$ for plasmas with positive (subplots (**a**,**b**)) and negative (subplots (**c**,**d**)) charged dusts. The left panels (subplots (**a**,**c**)) corresponding to the positive sign in ${U_{0}}^{2}$ (in Equation (16)) and the right ones (subplots (**b**,**d**)) are negative. Lines in each subplot represent different nonextensive *q* values, where the blue dotted lines represent q=1.2, the red solid lines represent q=1.0 and the green dashed lines represent q=0.8, respectively.

In Figure 3, we depict that the variation of η varies with the ion density ratio $\mu_{i}$. As we can see, for the left panels (subplots (a) and (c)), which correspond to the sign in $U_{0}^{2}$ is positive, η will increase with the growing of $\mu_{i}$. On the other hand, when the sign in $U_{0}^{2}$ is negative (subplots (b) and (d)), η will decrease as $\mu_{i}$ is increasing. When the dust is positively charged [the above panels (subplots (a) and (b))], η will have a decreasing trend. It is also seen that if the dust charge is positive, for different *q*, the changing of η is not significant. It means that the nonextensivity of the system on η is quite weak. However, when the dust is negatively charged, (subplots (c) and (d)), the nonextensivity will have a significant effect on η. When η has a growing trend, the larger the nonextensive parameter *q* is, the higher the value of η. It is the opposite when η has a decreasing trend, the larger the nonextensive parameter *q*, the lower the value of η. Therefore, the system’s nonextensivity will enhance the growing/decreasing of η. The nonextensive parameter *q* has the same effects as that of *A* or *B*.

**Figure 4:**
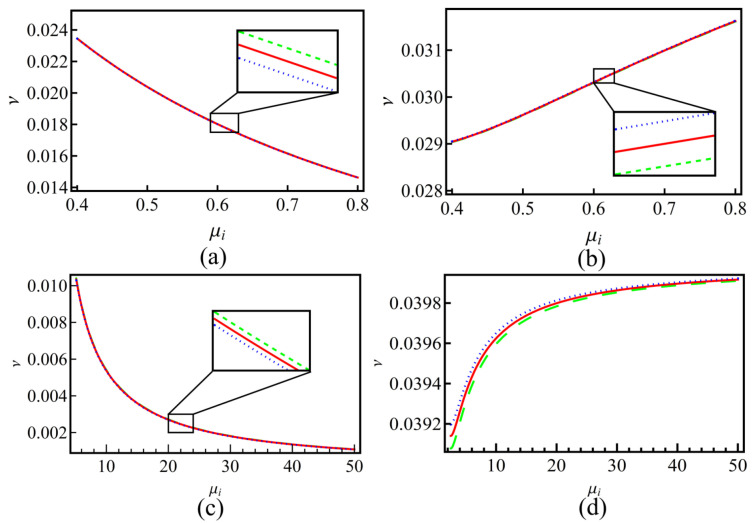
The ion–dust collisions coefficient ν versus the positive-to-negative ion density ratio $u_{i}$ for plasmas with positive (subplots (**a**,**b**)) and negative (subplots (**c**,**d**)) charged dust. Lines in each subplot represent different nonextensive *q* values that are the same as that in Figure 1. Other parameters are the same as Figure 1.

**Figure 5:**
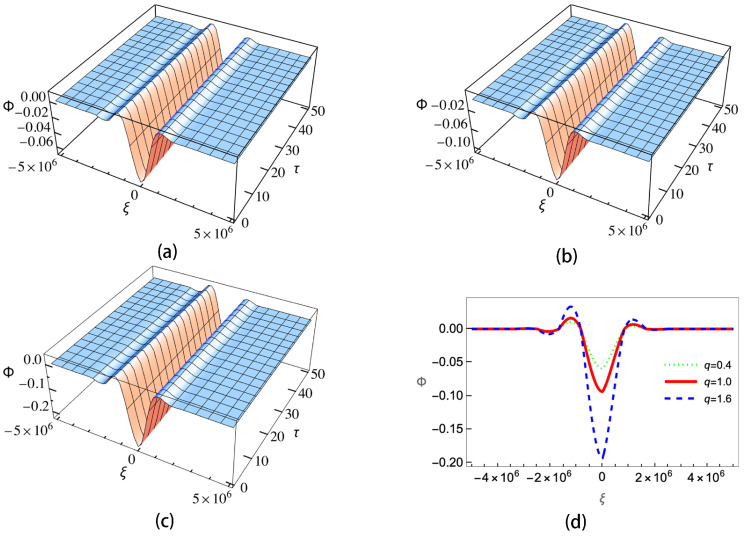
Wave evolutions with negative charged dust and positive sign in $U_{0}^{2}$ for (**a**) super-extensive (q=0.4), (**b**) Maxwellian (q=1.0), (**c**) sub-extensive electrons (q=1.6) and (**d**) wave amplitudes of the three cases at τ=50.

**Figure 6:**
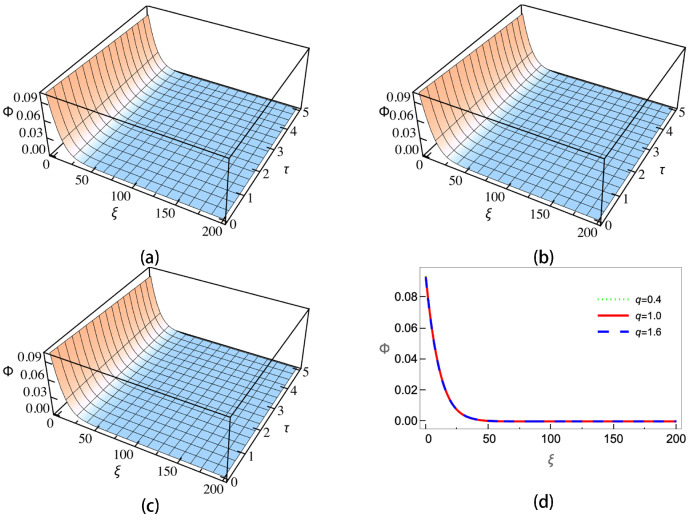
Wave profiles with negative charged dust and negative sign in $U_{0}^{2}$ for (**a**) super-extensive (q=0.4), (**b**) Maxwellian (q=1.0), (**c**) sub-extensive electrons (q=1.6) and (**d**) wave amplitudes of the three cases at τ=5.

**Figure 7:**
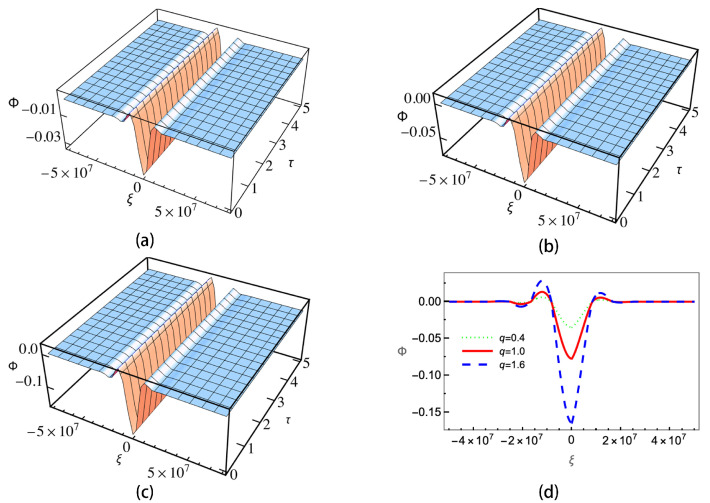
Wave evolution profiles with positive charged dust and positive sign in $U_{0}^{2}$ for (**a**) super-extensive (q=0.4), (**b**) Maxwellian (q=1.0), (**c**) sub-extensive electrons (q=1.6) and (**d**) wave amplitudes of the three cases at τ=5.

**Figure 8:**
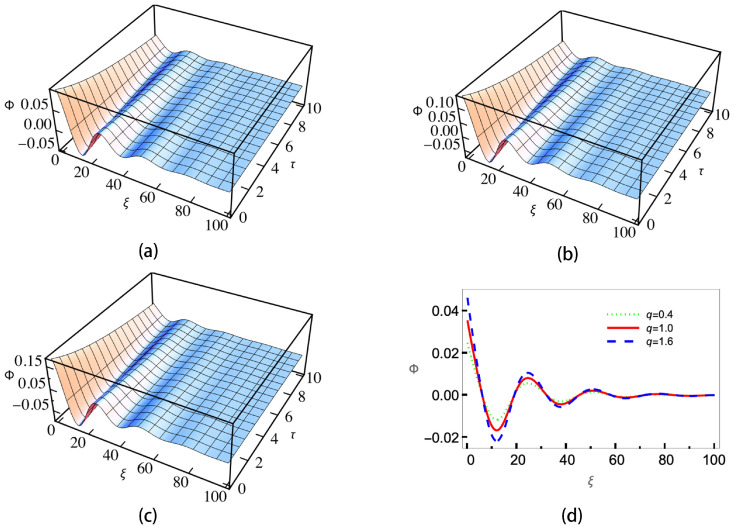
Wave evolution profiles with positive charged dust and negative sign in $U_{0}^{2}$ for (**a**) super-extensive (q=0.4), (**b**) Maxwellian (q=1), (**c**) sub-extensive electrons (q=1.6) and (**d**) wave amplitudes of the three cases at τ=10.

Figure 4 gives the ion–dust collisions coefficient ν versus the positive-to-negative ion density ratio $u_{i}$. As is shown, when the sign in $U_{0}^{2}$ is positive (subplots (a) and (c)), ν will decrease with the increasing of $\mu_{i}$. When the sign in $U_{0}^{2}$ is negative (subplots (b) and (d)), the trend is the opposite and ν will increase as $\mu_{i}$ is increasing. It can be also seen that for different *q*, the changing of ν is not significant except the case of negative charged dust with the sign in $U_{0}^{2}$ being negative. The enlarged view of the curves shows that the sub-extensive electrons (q>1) can enhance the growing/decreasing of ν, while the super-extensive ones (q<1) will weaken it. Equation (21) is a KdV Burgers-type equation with a damping term; it has no analytical solution. In order to investigate the effects of nonextensive electrons on the evolution of the wave, we numerically calculate Equation (21) under different conditions. The results are shown in Figure 5, Figure 6, Figure 7 and Figure 8.

Figure 5 corresponds to the case of negative charged dust and positive sign in $U_{0}^{2}$. Here, we let $u_{i}=1.5$. In this case, the value A>B≫η∼ν. Approximately, Equation (21), can be taken as a KdV equation. Therefore, we use a solitary wave solution as the initial condition $\Phi \left(\xi \right)=3v_{0}/A*sech^{2}\left[\xi /\left(2\sqrt{B/v_{0}}\right)\right]$, where $v_{0}=0.6$ is the wave speed. The wave evolutions for super-extensive (q<1), Maxwellian (q=1.0) and sub-extensive (q>1) electrons are shown in Figure 5a–c, respectively. Figure 5d gives the wave profiles of the three cases at τ=50. As shown in Figure 5, all three cases have damping effects due to ion–dust collisions and the amplitudes of Φ will decrease with τ. From Figure 5d, it is obvious that q=1.6 has the largest amplitude, while q=0.4 has the smallest one. Therefore, the nonextensivity will suppress the damping effect induced by ion–dust collisions.

Figure 6 depicts the wave evolutions of negatively charged dust and a negative sign in $U_{0}^{2}$. We let *u_i_* = 1.5 and the initial condition is set as $-\left(2v_{0}/B\right)Exp\left[\left(-\eta /2B\right)\xi \right]Cos\left(\sqrt{v_{0}/B}\xi \right)$, where $v_{0}=0.1$ is the wave speed. The numerical results of Equation (21) show that monotonic shock waves will be formed. Compared with the negative sign in the $U_{0}^{2}$ case, the three curves in Figure 6d almost coincide with each other. Therefore in this case, the nonextensive effects on wave evolution are quite weak.

Figure 7 gives the wave evolution profiles of positive charged dust and positive sign in $U_{0}^{2}$. Here we let *u_i_* = 5/7. The initial condition is set as $\Phi \left(\xi \right)=3v_{0}/A*sech^{2}\left[\xi /\left(2\sqrt{B/v_{0}}\right)\right]$, where $v_{0}=0.6$ is the wave speed. Similar to that of Figure 5, ion acoustic solitary waves will be formed. The sub-extensive case has the largest amplitude, and the super-extensive has the smallest one.

Figure 8 gives the wave evolution profiles of positive charged dust and negative sign in $U_{0}^{2}$. Here, as that in Figure 7, we let *u_i_* = 5/7. The initial condition is set as $-\left(2v_{0}/B\right)Exp\left[\left(-\eta /2B\right)\xi \right]Cos\left(\sqrt{v_{0}/B}\xi \right)$, where $v_{0}=0.1$ is the wave speed. Other parameters are the same as those of Figure 6. The numerical results show that oscillatory shock waves will be formed, and the system’s nonextensivity mainly affects the wave oscillatory amplitude. Compare with Figure 6, we can find that the wave speed $v_{0}$ will determine whether it is a oscillatory shock wave or monotonic one. When the speed is small, it is more likely to form a monotonic shock wave, while if the speed is large, a oscillatory shock wave will be formed [53].

## 5. Summary and Conclusions

In this paper, we studied the dust–ion acoustic waves in a collisional dusty plasma with negative ions. With the help of the reductive perturbation technique, we found that the wave evolution can be modeled by the KdV Burgers type equation with a damping term that is related to the ion–dust collisions. This plasma system has four types of ion acoustic waves, fast/slow mode with positive/negative ions, respectively. We analyze the effects of nonextensive electrons on wave evolution through numerical methods. Our results show that the nonextensive electrons will affect the wave amplitude. If the wave has a growing trend, the sub-extensive electrons (q>1) will enhance the wave amplitude, while the super-extensive ones (q<1) will weaken it. If the wave has a decreasing trend, the sub-extensive electrons will enhance the wave’s decreasing trend, while the super-extensive ones will weaken it. Furthermore, we expect that our investigation will be helpful for future investigations on dust–ion acoustic solitary and shock waves.

## Data Availability

The data that support the findings of this study are available from the corresponding author upon reasonable request.

## Abbreviations

The following abbreviations are used in this manuscript:

IAW	Ion Acoustic Wave
DIAW	Dust–Ion Acoustic Wave
IASWS	Ion Acoustic Solitary Wave and Shocks
KdVB	Korteweg–de Vries Burgers

## References

[1] Lee M.J., Jung Y.D. (2019). Temperature effects on the propagation and Landau damping of the dust surface waves. Phys. Plasmas.

[2] Bilal M., Rehman A.U., Mahmood S., Shahzad M.A., Sarfraz M. (2023). Landau damping of ion-acoustic waves with simultaneous effects of non-extensivity and non-thermality in the presence of hybrid Cairns-Tsallis distributed electrons. Contrib. Plasma Phys..

[3] Beving L.P., Hopkins M.M., Baalrud S.D. (2021). Simulations of ion heating due to ion-acoustic instabilities in presheaths. Phys. Plasmas.

[4] Hellinger P., Trávníček P., Menietti J.D. (2004). Effective collision frequency due to ion-acoustic instability: Theory and simulations. Geophys. Res. Lett..

[5] Khalid M. (2022). Oblique ion-acoustic solitary waves in anisotropic plasma with Tsallis distribution. Europhys. Lett..

[6] Madhukalya B., Das R., Hosseini K., Baleanu D., Salahshour S. (2023). Small amplitude ion-acoustic solitary waves in a magnetized ion-beam plasma under the effect of ion and beam temperatures. Euro. Phys. J. Plus.

[7] Biskamp D., Parkinson D. (1970). Ion Acoustic Shock Waves. Phys. Fluids.

[8] Ikezi H., Taylor R., Baker D. (1970). Formation and interaction of ion-acoustic solitions. Phys. Rev. Lett..

[9] Das G.C. (1979). Ion-acoustic solutions and shock waves in multicomponent plasmas. Plasma Phys..

[10] Shukla P.K., Silin V.P. (1992). Dust ion-acoustic wave. Phys. Scr..

[11] Saleem H., Shan S.A. (2021). Solar wind interaction with dusty plasma produces electrostatic instabilities and solitons. Astrophys. Space Sci..

[12] Bansal S., Aggarwal M., Gill T.S. (2020). Nonplanar ion acoustic waves in dusty plasma with two temperature electrons: Application to Saturn’s E ring. Phys. Plasmas.

[13] Hirashita H. (2012). Dust growth in the interstellar medium: How do accretion and coagulation interplay?. Mon. Not. R. Astron. Soc..

[14] Mann I., Gunnarsdottir T., Häggström I., Eren S., Tjulin A., Myrvang M., Rietveld M., Dalin P., Jozwicki D., Trollvik H. (2019). Radar studies of ionospheric dusty plasma phenomena. Contrib. Plasma Phys..

[15] Merlino R. (2021). Dusty plasmas: From Saturn’s rings to semiconductor processing devices. Adv. Phys. X.

[16] Long J.M., Ou J. (2022). Dust particle surface potential in fusion plasma with supra-thermal electrons. Phys. Plasmas.

[17] El-Labany S., Moslem W.M., Mowafy A. (2003). Effects of trapped electron temperature, dust charge variations, and grain radius on the existence of the dust-ion-acoustic waves. Phys. Plasmas.

[18] Vranješ J., Pandey B., Poedts S. (2002). Ion–acoustic waves in dusty plasma with charge fluctuations. Phys. Plasmas.

[19] El-Labany S., El-Siragy N., El-Taibany W., El-Shamy E., Behery E. (2010). Linear and nonlinear quantum dust ion acoustic wave with dust size distribution effect. Phys. Plasmas.

[20] Chutia B., Deka T., Bailung Y., Sharma D., Sharma S., Bailung H. (2021). Spatiotemporal evolution of a self-excited dust density wave in a nanodusty plasma under strong Havnes effect. Phys. Plasmas.

[21] Sharma R., Bhardwaj S., Dhiman J.S. (2020). Effects of dust temperature and radiative heat-loss functions on the magnetogravitational instability of viscoelastic dusty plasma. Astrophys. Space Sci..

[22] Gan Y., Xu A., Zhang G., Succi S. (2015). Discrete Boltzmann modeling of multiphase flows: Hydrodynamic and thermodynamic non-equilibrium effects. Soft Matter.

[23] Gan Y., Xu A., Zhang G., Zhang Y., Succi S. (2018). Discrete Boltzmann trans-scale modeling of high-speed compressible flows. Phys. Rev. E.

[24] Gan Y.B., Xu A.G., Zhang G.C., Lin C.D., Lai H.L., Liu Z.P. (2019). Nonequilibrium and morphological characterizations of Kelvin–Helmholtz instability in compressible flows. Front. Phys..

[25] Gan Y., Xu A., Lai H., Li W., Sun G., Succi S. (2022). Discrete Boltzmann multi-scale modelling of non-equilibrium multiphase flows. J. Fluid Mech..

[26] Liu Z., Liu L., Du J. (2009). A nonextensive approach for the instability of current-driven ion-acoustic waves in space plasmas. Phys. Plasmas.

[27] Liu Z., Du J. (2009). Dust acoustic instability driven by drifting ions and electrons in the dust plasma with Lorentzian kappa distribution. Phys. Plasmas.

[28] Liu Z., Song J., Xu A., Zhang Y., Xie K. (2023). Discrete Boltzmann modeling of plasma shock wave. Proc. IMechE Part C J. Mech. Eng. Sci..

[29] Vasyliunas V.M. (1968). A survey of low-energy electrons in the evening sector of the magnetosphere with OGO 1 and OGO 3. J. Geophys. Res..

[30] Leubner M.P. (2002). A Nonextensive Entropy Approach to Kappa-Distributions. Astrophys. Space Sci..

[31] Livadiotis G., McComas D.J. (2009). Beyond kappa distributions: Exploiting Tsallis statistical mechanics in space plasmas. J. Geophys. Res. Space Phys..

[32] Livadiotis G., McComas D. (2010). Exploring transitions of space plasmas out of equilibrium. Astrophys. J..

[33] Livadiotis G., McComas D. (2011). Invariant kappa distribution in space plasmas out of equilibrium. Astrophys. J..

[34] Tsallis C. (1988). Possible generalization of Boltzmann-Gibbs statistics. J. Stat. Phys..

[35] Du J. (2006). What does the nonextensive parameter stand for in self-gravitating systems?. Astrophys. Space Sci..

[36] Deppman A., Megías E.P., Menezes D. (2020). Fractal Structures of Yang–Mills Fields and Non-Extensive Statistics: Applications to High Energy Physics. Physics.

[37] Megías E., Timóteo V., Gammal A., Deppman A. (2022). Bose–Einstein condensation and non-extensive statistics for finite systems. Phys. A.

[38] Du J. (2004). Nonextensivity in nonequilibrium plasma systems with Coulombian long-range interactions. Phys. Lett. A.

[39] Du J. (2004). The nonextensive parameter and Tsallis distribution for self-gravitating systems. Europhys. Lett..

[40] Du J. (2007). Nonextensivity and the power-law distributions for the systems with self-gravitating long-range interactions. Astrophys. Space Sci..

[41] Yu H., Du J. (2017). The nonextensive parameter for the rotating astrophysical systems with power-law distributions. Europhys. Lett..

[42] Gong J., Du J. (2012). Dust charging processes in the nonequilibrium dusty plasma with nonextensive power-law distribution. Phys. Plasmas.

[43] Gong J., Liu Z., Du J. (2012). Dust-acoustic waves and stability in the permeating dusty plasma. II. Power-law distributions. Phys. Plasmas.

[44] Du J. (2013). Transport coefficients in Lorentz plasmas with the power-law kappa-distribution. Phys. Plasmas.

[45] Wang L., Du J. (2017). The diffusion of charged particles in the weakly ionized plasma with power-law kappa-distributions. Phys. Plasmas.

[46] Wang Y., Du J. (2018). The viscosity of charged particles in the weakly ionized plasma with power-law distributions. Phys. Plasmas.

[47] Nicolaou G., Livadiotis G. (2020). Statistical Uncertainties of Space Plasma Properties Described by Kappa Distributions. Entropy.

[48] Lima J.A.S., Silva R., Santos J. (2000). Plasma oscillations and nonextensive statistics. Phys. Rev. E.

[49] Younsi S., Tribeche M. (2010). Arbitrary amplitude electron-acoustic solitary waves in the presence of excess superthermal electrons. Astrophys. Space Sci..

[50] El-Awady E., Moslem W. (2011). On a plasma having nonextensive electrons and positrons: Rogue and solitary wave propagation. Phys. Plasmas.

[51] Yasmin S., Asaduzzaman M., Mamun A. (2013). Dust ion-acoustic shock waves in nonextensive dusty plasma. Astrophys. Space Sci..

[52] Misra A.P., Adhikary N.C., Shukla P.K. (2012). Ion-acoustic solitary waves and shocks in a collisional dusty negative-ion plasma. Phys. Rev. E.

[53] Pakzad H.R., Javidan K. (2009). Dust acoustic solitary and shock waves in strongly coupled dusty plasmas with nonthermal ions. Pramana.

